# [(1*R*,3*S*)-6,7-Dimeth­oxy-1-phenyl-1,2,3,4-tetra­hydro­isoquinolin-3-yl]methanol 2.33-hydrate

**DOI:** 10.1107/S1600536811006052

**Published:** 2011-02-23

**Authors:** Sai Kumar Chakka, Michael G. McKay, Thavendran Govender, Hendrik G. Kruger, Glenn E. M. Maguire

**Affiliations:** a School of Chemistry, University of KwaZulu-Natal, Durban 4000, South Africa; b School of Pharmacy and Pharmacology, University of KwaZulu-Natal, Durban, South Africa

## Abstract

The title compound, C_18_H_21_NO_3_·2.33H_2_O, is the fourth reported member in a series of (1*R*,3*S*)-6,7-dimeth­oxy-1-phenyl-1,2,3,4-tetra­hydro­isoquinoline derivatives used in catalysis as ligands (or their precursors). The *N*-heterocycle in the structure adopts a half-chair conformation. The dihedral angle between the benzene rings is 77.29 (13)°. There are three ill-resolved water molecules of crystallization in the structure (one of them rotationally disordered about a threefold axis) involved in short contacts probably due to hydrogen bonding.

## Related literature

For the synthesis of the ligand, see: Chakka *et al.* (2009 ▶). For the Henry reaction, see: Kawthekar *et al.* (2010 ▶). For similar structures, see: Naicker *et al.* (2009 ▶, 2010*a* ▶,*b* ▶); Chakka *et al.* (2010 ▶).
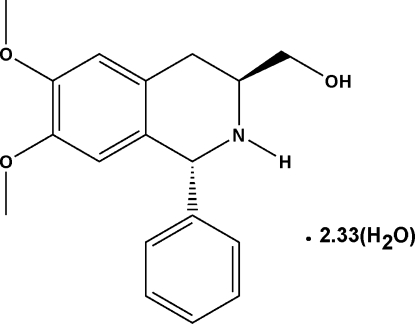


## Experimental

### 

#### Crystal data

C_18_H_21_NO_3_·2.33H_2_O
*M* *_r_* = 341.39
Trigonal, 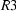

*a* = 27.950 (2) Å
*c* = 5.8035 (5) Å
*V* = 3926 (2) Å^3^
*Z* = 9
Mo *K*α radiation
μ = 0.10 mm^−1^
*T* = 173 K
0.13 × 0.12 × 0.09 mm

#### Data collection

Bruker Kappa DUO APEXII CCD diffractometer
10224 measured reflections
4340 independent reflections
3707 reflections with *I* > 2σ(*I*)
*R*_int_ = 0.022

#### Refinement

*R*[*F* ^2^ > 2σ(*F* ^2^)] = 0.056
*wR*(*F* ^2^) = 0.162
*S* = 1.06
4340 reflections
226 parameters
3 restraints
H atoms treated by a mixture of independent and constrained refinement
Δρ_max_ = 0.60 e Å^−3^
Δρ_min_ = −0.30 e Å^−3^

### 

Data collection: *APEX2* (Bruker, 2006 ▶); cell refinement: *SAINT* (Bruker, 2006 ▶); data reduction: *SAINT*; program(s) used to solve structure: *SHELXS97* (Sheldrick, 2008 ▶); program(s) used to refine structure: *SHELXL97* (Sheldrick, 2008 ▶); molecular graphics: *OLEX2* (Dolomanov *et al.*, 2009 ▶); software used to prepare material for publication: *SHELXL97*.

## Supplementary Material

Crystal structure: contains datablocks I, global. DOI: 10.1107/S1600536811006052/bg2388sup1.cif

Structure factors: contains datablocks I. DOI: 10.1107/S1600536811006052/bg2388Isup2.hkl

Additional supplementary materials:  crystallographic information; 3D view; checkCIF report

## Figures and Tables

**Table 1:** Hydrogen-bond geometry (Å, °)

*D*—H⋯*A*	*D*—H	H⋯*A*	*D*⋯*A*	*D*—H⋯*A*
O3—H3*O*⋯O1*W*	0.99 (4)	1.95 (4)	2.917 (6)	164 (5)

**Fig. 1.**
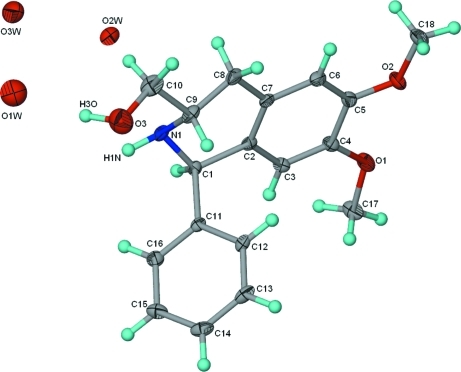
Molecular structure of (I) showing numbering scheme. All non-hydrogen atoms except O1W are shown as ellipsoids with probability level of 30%.

## supplementary crystallographic information

### Comment

Heterocyclic rings play key roles in a number of areas of organic and inorganic chemistry. As part of an ongoing study employing (1*R*,3*S*)-6,7-dimethoxy-1-phenyl-1,2,3,4-tetrahydroisoquinoline based metal complexes as catalysts in asymmetric hydride transfer reactions (Chakka *et al.*, 2009) and the Henry reaction (Kawthekar *et al.*, 2010)  we synthesized the title compound. The absolute sterochemistry of the diastereomer was confirmed by NMR studies as *R,S* at C1 and C9. The primary alcohol group displays hydrogen bonding (O3—H3O···O1W)(2.917 (6) A) (Fig 1.).

The first (1*R*,3*S*)-6,7-dimethoxy-1-phenyl-1,2,3,4-tetrahydroisoquinoline structure we reported (Naicker *et al.*, 2009) had an ester functionality at the C9 position and its *N-*heterocycle revealed a half boat conformation. For the title compound the *N-*heterocycle adopts a half chair conformation, as it does in the remaining two related structures that we have communicated (Naicker *et al.*, 2010*a*; Naicker *et al.*, 2010*b*).

There are in the structure of the title compound a number of short O···O contacts involving the crystal water molecules (one of them, O1W, rotationally disordered on a three fold axis), probably due to hydrogen bonding but which could not be considered in detail because of the impossibility to find the water H atoms.

### Experimental

A solution of amino ester (0.5 g, 1.5 mmol) in dry THF (20 ml) (Chakka *et al.*, 2009) was added dropwise to a suspension of LiAlH_4_ (0.18, 4.5 mmol) in dry THF (20 ml) under N_2_ atmosphere at 0 °C. The mixture was stirred at 0 °C for 2 h, and the reaction was monitored with TLC in hexane/ethyl acetate (50/50, *R*_f_ = 1/2). Excess lithium aluminium hydride was quenched with saturated sodium sulfate solution at 0 °C. The reaction mixture was filtered and the solid was washed with THF (20 ml). The solvent was evaporated to dryness, ethyl acetate (20 ml) was added, washed with water (2 × 5 ml), the organic layer was separated and dried over anhydrous MgSO_4_ to afford the crude amino alcohol. This was purified by gradient column chromatography; solvent A: 10:90 saturated ammonia in MeOH:DCM and solvent B: 2:98 MeOH:DCM to yield 0.33 g (70% yield) of the pale yellow target compound. m.p.= 388–390 K Crystals apt for x-ray diffraction were grown in methanol, at room temperature. The water molecules in the crystal were probably due to contamination of the solvent.

### Refinement

There is one main molecule and two and one-third water molecules in the asymmetric unit. Water molecule O1W is disordered on a site of higher rotational (threefold) symmetry than its own (twofold). It has accordingly a high temperature factor (*U*_iso_ = 0.0959), for what it was refined isotropically. All hydrogen atoms attached to carbon were positioned geometrically with C—H = 0.95 - 1.00 Å and refined as riding on their parent atoms. The hydrogen atoms H3O and H1N were located in a difference electron density map and refined with simple bond length constraints. In all cases *U*_iso_ (H) = 1.2 - 1.5 *U*_eq_ (Host). In spite of the low temperature data, the hydrogen atoms on the three water molecules could not be found and therefore were excluded from the final model.

### Crystal data

**Table:**

C_18_H_21_NO_3_·2.33H_2_O	*D*_x_ = 1.299 Mg m^−^^3^
*M**_r_* = 341.39	Mo *K*α radiation, λ = 0.71073 Å
Trigonal, *R*3	Cell parameters from 10224 reflections
Hall symbol: R 3	θ = 2.5–28.3°
*a* = 27.950 (2) Å	µ = 0.10 mm^−^^1^
*c* = 5.8035 (5) Å	*T* = 173 K
*V* = 3926 (2) Å^3^	Needle, colourless
*Z* = 9	0.13 × 0.12 × 0.09 mm
*F*(000) = 1650	

### Data collection

**Table:**

Bruker Kappa DUO APEXII CCD diffractometer	3707 reflections with *I* > 2σ(*I*)
Radiation source: fine-focus sealed tube	*R*_int_ = 0.022
graphite	θ_max_ = 28.3°, θ_min_ = 2.5°
0.5° φ scans and ω	*h* = −37→37
10224 measured reflections	*k* = −31→37
4340 independent reflections	*l* = −7→7

### Refinement

**Table:**

Refinement on *F*^2^	Primary atom site location: structure-invariant direct methods
Least-squares matrix: full	Secondary atom site location: difference Fourier map
*R*[*F*^2^ > 2σ(*F*^2^)] = 0.056	Hydrogen site location: inferred from neighbouring sites
*wR*(*F*^2^) = 0.162	H atoms treated by a mixture of independent and constrained refinement
*S* = 1.06	*w* = 1/[σ^2^(*F*_o_^2^) + (0.0985*P*)^2^ + 2.1271*P*] where *P* = (*F*_o_^2^ + 2*F*_c_^2^)/3
4340 reflections	(Δ/σ)_max_ < 0.001
226 parameters	Δρ_max_ = 0.60 e Å^−^^3^
3 restraints	Δρ_min_ = −0.30 e Å^−^^3^

### Special details

**Table:**

Experimental. Half sphere of data collected using *SAINT* strategy (Bruker, 2006). Crystal to detector distance = 50 mm; combination of φ and ω scans of 0.5°, 60 s per °, 2 iterations.
Geometry. All e.s.d.'s (except the e.s.d. in the dihedral angle between two l.s. planes) are estimated using the full covariance matrix. The cell e.s.d.'s are taken into account individually in the estimation of e.s.d.'s in distances, angles and torsion angles; correlations between e.s.d.'s in cell parameters are only used when they are defined by crystal symmetry. An approximate (isotropic) treatment of cell e.s.d.'s is used for estimating e.s.d.'s involving l.s. planes.
Refinement. Refinement of *F*^2^ against ALL reflections. The weighted *R*-factor *wR* and goodness of fit *S* are based on *F*^2^, conventional *R*-factors *R* are based on *F*, with *F* set to zero for negative *F*^2^. The threshold expression of *F*^2^ > σ(*F*^2^) is used only for calculating *R*-factors(gt) *etc*. and is not relevant to the choice of reflections for refinement. *R*-factors based on *F*^2^ are statistically about twice as large as those based on *F*, and *R*- factors based on ALL data will be even larger.

### Fractional atomic coordinates and isotropic or equivalent isotropic displacement parameters (Å^2^)

**Table:**

	*x*	*y*	*z*	*U*_iso_*/*U*_eq_	
O1	0.91196 (8)	0.15356 (9)	0.2897 (4)	0.0468 (5)	
O2	0.95248 (8)	0.22297 (9)	0.6202 (3)	0.0484 (5)	
O3	0.67745 (12)	0.27007 (15)	0.4378 (6)	0.0809 (9)	
H3O	0.675 (2)	0.287 (2)	0.291 (5)	0.097*	
O1W	0.6667	0.3333	0.0620 (11)	0.0959 (17)*	
O2W	0.82161 (9)	0.36012 (8)	0.0053 (3)	0.0477 (5)	
O3W	0.76609 (13)	0.41438 (12)	−0.0962 (6)	0.0806 (8)	
N1	0.75539 (10)	0.24710 (8)	0.1792 (4)	0.0370 (5)	
H1N	0.7216 (8)	0.2411 (13)	0.108 (5)	0.043*	
C1	0.76741 (9)	0.20211 (9)	0.1292 (4)	0.0285 (4)	
H1	0.7785	0.2059	−0.0366	0.034*	
C2	0.81654 (9)	0.20906 (9)	0.2690 (4)	0.0299 (4)	
C3	0.84111 (9)	0.17779 (10)	0.2090 (4)	0.0327 (5)	
H3	0.8270	0.1529	0.0825	0.039*	
C4	0.88544 (10)	0.18267 (10)	0.3311 (4)	0.0348 (5)	
C5	0.90748 (11)	0.22030 (11)	0.5167 (4)	0.0369 (5)	
C6	0.88280 (12)	0.25024 (10)	0.5776 (4)	0.0375 (5)	
H6	0.8969	0.2749	0.7045	0.045*	
C7	0.83662 (10)	0.24501 (9)	0.4548 (4)	0.0314 (5)	
C8	0.81126 (12)	0.27861 (10)	0.5265 (4)	0.0399 (6)	
H8A	0.8346	0.3171	0.4722	0.048*	
H8B	0.8096	0.2793	0.6968	0.048*	
C9	0.75403 (13)	0.25544 (11)	0.4300 (4)	0.0411 (6)	
H9	0.7287	0.2193	0.5058	0.049*	
C10	0.73288 (16)	0.29565 (15)	0.4714 (7)	0.0594 (8)	
H10A	0.7417	0.3100	0.6310	0.071*	
H10B	0.7516	0.3274	0.3646	0.071*	
C11	0.71644 (9)	0.14513 (9)	0.1564 (4)	0.0280 (4)	
C12	0.70780 (10)	0.11199 (10)	0.3506 (4)	0.0337 (5)	
H12	0.7343	0.1252	0.4715	0.040*	
C13	0.66148 (12)	0.06060 (11)	0.3683 (5)	0.0433 (6)	
H13	0.6562	0.0387	0.5013	0.052*	
C14	0.62243 (11)	0.04056 (11)	0.1934 (6)	0.0463 (7)	
H14	0.5909	0.0047	0.2051	0.056*	
C15	0.62937 (11)	0.07295 (11)	0.0015 (5)	0.0451 (6)	
H15	0.6024	0.0597	−0.1175	0.054*	
C16	0.67646 (10)	0.12540 (10)	−0.0156 (4)	0.0374 (5)	
H16	0.6811	0.1478	−0.1464	0.045*	
C17	0.88839 (12)	0.11055 (14)	0.1231 (6)	0.0509 (7)	
H17A	0.9112	0.0931	0.1098	0.076*	
H17B	0.8868	0.1260	−0.0264	0.076*	
H17C	0.8510	0.0829	0.1711	0.076*	
C18	0.97424 (13)	0.25805 (14)	0.8174 (5)	0.0533 (8)	
H18A	1.0064	0.2566	0.8759	0.080*	
H18B	0.9458	0.2455	0.9375	0.080*	
H18C	0.9854	0.2961	0.7738	0.080*	

### Atomic displacement parameters (Å^2^)

**Table:**

	*U*^11^	*U*^22^	*U*^33^	*U*^12^	*U*^13^	*U*^23^
O1	0.0351 (10)	0.0572 (12)	0.0562 (11)	0.0292 (9)	−0.0095 (8)	−0.0163 (9)
O2	0.0420 (10)	0.0542 (12)	0.0480 (11)	0.0233 (9)	−0.0183 (8)	−0.0117 (9)
O3	0.0626 (17)	0.092 (2)	0.095 (2)	0.0430 (15)	−0.0005 (15)	−0.0043 (17)
O2W	0.0541 (12)	0.0409 (10)	0.0489 (11)	0.0243 (9)	0.0054 (9)	0.0036 (8)
O3W	0.0695 (17)	0.0625 (16)	0.115 (2)	0.0372 (14)	−0.0191 (16)	−0.0032 (15)
N1	0.0478 (12)	0.0286 (10)	0.0405 (11)	0.0235 (9)	−0.0004 (9)	0.0020 (8)
C1	0.0311 (11)	0.0278 (10)	0.0258 (9)	0.0141 (9)	0.0038 (8)	0.0054 (8)
C2	0.0331 (11)	0.0253 (10)	0.0273 (10)	0.0117 (9)	0.0072 (8)	0.0055 (8)
C3	0.0296 (11)	0.0341 (11)	0.0305 (11)	0.0130 (9)	0.0028 (8)	−0.0035 (8)
C4	0.0294 (11)	0.0373 (12)	0.0332 (11)	0.0131 (10)	0.0024 (9)	−0.0009 (9)
C5	0.0372 (12)	0.0357 (12)	0.0328 (11)	0.0143 (10)	−0.0036 (9)	0.0022 (9)
C6	0.0500 (14)	0.0312 (11)	0.0271 (10)	0.0170 (11)	−0.0019 (10)	−0.0004 (9)
C7	0.0410 (12)	0.0218 (10)	0.0298 (10)	0.0145 (9)	0.0017 (9)	0.0031 (8)
C8	0.0631 (17)	0.0325 (12)	0.0303 (11)	0.0284 (12)	−0.0007 (11)	−0.0016 (9)
C9	0.0557 (16)	0.0351 (12)	0.0414 (13)	0.0295 (12)	0.0118 (11)	0.0061 (10)
C10	0.063 (2)	0.0548 (18)	0.072 (2)	0.0387 (17)	0.0177 (16)	0.0012 (15)
C11	0.0309 (11)	0.0252 (10)	0.0307 (10)	0.0162 (9)	0.0070 (8)	0.0024 (8)
C12	0.0377 (12)	0.0328 (11)	0.0374 (12)	0.0227 (10)	0.0091 (9)	0.0091 (9)
C13	0.0474 (14)	0.0320 (12)	0.0557 (15)	0.0237 (11)	0.0239 (12)	0.0150 (11)
C14	0.0347 (13)	0.0298 (12)	0.0675 (18)	0.0108 (10)	0.0190 (13)	−0.0014 (11)
C15	0.0336 (12)	0.0370 (13)	0.0546 (16)	0.0101 (11)	0.0033 (11)	−0.0077 (11)
C16	0.0375 (12)	0.0350 (12)	0.0374 (12)	0.0165 (10)	0.0038 (10)	0.0016 (10)
C17	0.0382 (14)	0.0600 (17)	0.0640 (19)	0.0317 (14)	−0.0060 (12)	−0.0230 (14)
C18	0.0462 (16)	0.0548 (17)	0.0419 (14)	0.0126 (13)	−0.0158 (12)	−0.0037 (12)

### Geometric parameters (Å, °)

**Table:**

O1—C4	1.368 (3)	C8—H8B	0.9900
O1—C17	1.422 (3)	C9—C10	1.528 (4)
O2—C5	1.362 (3)	C9—H9	1.0000
O2—C18	1.430 (3)	C10—H10A	0.9900
O3—C10	1.357 (5)	C10—H10B	0.9900
O3—H3O	0.99 (4)	C11—C16	1.390 (3)
N1—C9	1.477 (3)	C11—C12	1.401 (3)
N1—C1	1.484 (3)	C12—C13	1.375 (4)
N1—H1N	0.97 (3)	C12—H12	0.9500
C1—C2	1.521 (3)	C13—C14	1.387 (5)
C1—C11	1.524 (3)	C13—H13	0.9500
C1—H1	1.0000	C14—C15	1.386 (4)
C2—C7	1.387 (3)	C14—H14	0.9500
C2—C3	1.399 (3)	C15—C16	1.401 (4)
C3—C4	1.374 (3)	C15—H15	0.9500
C3—H3	0.9500	C16—H16	0.9500
C4—C5	1.414 (3)	C17—H17A	0.9800
C5—C6	1.370 (4)	C17—H17B	0.9800
C6—C7	1.417 (4)	C17—H17C	0.9800
C6—H6	0.9500	C18—H18A	0.9800
C7—C8	1.491 (3)	C18—H18B	0.9800
C8—C9	1.502 (4)	C18—H18C	0.9800
C8—H8A	0.9900		
			
C4—O1—C17	117.5 (2)	N1—C9—H9	109.6
C5—O2—C18	117.1 (2)	C8—C9—H9	109.6
C10—O3—H3O	102 (3)	C10—C9—H9	109.6
C9—N1—C1	111.16 (18)	O3—C10—C9	110.5 (3)
C9—N1—H1N	110 (2)	O3—C10—H10A	109.6
C1—N1—H1N	112.6 (19)	C9—C10—H10A	109.6
N1—C1—C2	111.10 (19)	O3—C10—H10B	109.6
N1—C1—C11	112.09 (18)	C9—C10—H10B	109.6
C2—C1—C11	113.00 (17)	H10A—C10—H10B	108.1
N1—C1—H1	106.7	C16—C11—C12	118.5 (2)
C2—C1—H1	106.7	C16—C11—C1	119.09 (19)
C11—C1—H1	106.7	C12—C11—C1	122.4 (2)
C7—C2—C3	119.9 (2)	C13—C12—C11	120.8 (3)
C7—C2—C1	121.3 (2)	C13—C12—H12	119.6
C3—C2—C1	118.74 (19)	C11—C12—H12	119.6
C4—C3—C2	120.6 (2)	C12—C13—C14	120.5 (2)
C4—C3—H3	119.7	C12—C13—H13	119.8
C2—C3—H3	119.7	C14—C13—H13	119.8
O1—C4—C3	125.5 (2)	C15—C14—C13	120.0 (2)
O1—C4—C5	114.3 (2)	C15—C14—H14	120.0
C3—C4—C5	120.2 (2)	C13—C14—H14	120.0
O2—C5—C6	125.9 (2)	C14—C15—C16	119.5 (3)
O2—C5—C4	115.1 (2)	C14—C15—H15	120.3
C6—C5—C4	119.1 (2)	C16—C15—H15	120.3
C5—C6—C7	121.2 (2)	C11—C16—C15	120.8 (2)
C5—C6—H6	119.4	C11—C16—H16	119.6
C7—C6—H6	119.4	C15—C16—H16	119.6
C2—C7—C6	118.9 (2)	O1—C17—H17A	109.5
C2—C7—C8	121.7 (2)	O1—C17—H17B	109.5
C6—C7—C8	119.4 (2)	H17A—C17—H17B	109.5
C7—C8—C9	111.3 (2)	O1—C17—H17C	109.5
C7—C8—H8A	109.4	H17A—C17—H17C	109.5
C9—C8—H8A	109.4	H17B—C17—H17C	109.5
C7—C8—H8B	109.4	O2—C18—H18A	109.5
C9—C8—H8B	109.4	O2—C18—H18B	109.5
H8A—C8—H8B	108.0	H18A—C18—H18B	109.5
N1—C9—C8	109.2 (2)	O2—C18—H18C	109.5
N1—C9—C10	108.7 (2)	H18A—C18—H18C	109.5
C8—C9—C10	110.2 (2)	H18B—C18—H18C	109.5
			
C9—N1—C1—C2	−48.0 (3)	C1—C2—C7—C8	−0.1 (3)
C9—N1—C1—C11	79.5 (3)	C5—C6—C7—C2	−0.4 (3)
N1—C1—C2—C7	14.4 (3)	C5—C6—C7—C8	−179.7 (2)
C11—C1—C2—C7	−112.6 (2)	C2—C7—C8—C9	18.5 (3)
N1—C1—C2—C3	−166.43 (19)	C6—C7—C8—C9	−162.2 (2)
C11—C1—C2—C3	66.6 (3)	C1—N1—C9—C8	68.4 (2)
C7—C2—C3—C4	−0.6 (3)	C1—N1—C9—C10	−171.4 (2)
C1—C2—C3—C4	−179.8 (2)	C7—C8—C9—N1	−51.1 (3)
C17—O1—C4—C3	−7.1 (4)	C7—C8—C9—C10	−170.5 (2)
C17—O1—C4—C5	173.1 (3)	N1—C9—C10—O3	75.4 (3)
C2—C3—C4—O1	178.9 (2)	C8—C9—C10—O3	−165.0 (3)
C2—C3—C4—C5	−1.2 (4)	N1—C1—C11—C16	76.9 (3)
C18—O2—C5—C6	3.4 (4)	C2—C1—C11—C16	−156.7 (2)
C18—O2—C5—C4	−176.4 (2)	N1—C1—C11—C12	−102.1 (2)
O1—C4—C5—O2	2.0 (3)	C2—C1—C11—C12	24.3 (3)
C3—C4—C5—O2	−177.9 (2)	C16—C11—C12—C13	1.5 (3)
O1—C4—C5—C6	−177.9 (2)	C1—C11—C12—C13	−179.5 (2)
C3—C4—C5—C6	2.3 (4)	C11—C12—C13—C14	0.1 (4)
O2—C5—C6—C7	178.7 (2)	C12—C13—C14—C15	−1.5 (4)
C4—C5—C6—C7	−1.5 (4)	C13—C14—C15—C16	1.2 (4)
C3—C2—C7—C6	1.4 (3)	C12—C11—C16—C15	−1.8 (4)
C1—C2—C7—C6	−179.4 (2)	C1—C11—C16—C15	179.2 (2)
C3—C2—C7—C8	−179.2 (2)	C14—C15—C16—C11	0.5 (4)

### Hydrogen-bond geometry (Å, °)

**Table:**

*D*—H···*A*	*D*—H	H···*A*	*D*···*A*	*D*—H···*A*
O3—H3O···O1W	0.99 (4)	1.95 (4)	2.917 (6)	164 (5)
